# Brass‐mesh‐bolus–Radiation safety analysis and activation characterization following high energy photon treatment

**DOI:** 10.1002/acm2.70174

**Published:** 2025-07-14

**Authors:** Richard C. Mallory, Kyle Woods, Kiernan McCullough

**Affiliations:** ^1^ Colorado Associates in Medical Physics Colorado Springs Colorado USA

**Keywords:** activation products, brass‐mesh‐bolus, radiation protection, therapist safety

## Abstract

**Purpose:**

Brass‐mesh‐bolus (BMB) has been proposed as an alternative to water‐equivalent bolus due to its ease of setup and conformality to patient contour. In high‐energy beams, BMB can become radioactive and pose a potential exposure risk to therapists from frequent exposure while handling. Very little research has been performed on the method of activation and dose from clinically realistic activation of BMB, necessitating the need for further investigation.

**Methods:**

To determine the expected activation of the BMB, products via neutron‐capture were calculated using thermal‐neutron cross‐section tables, as has been performed in previous literature. A novel consideration is to include the photo‐neutron activation components. Measurements were performed using an NaI‐scintillator after BMB irradiation, assessing its activity for both in‐field and out‐of‐field activation. The collected gamma spectrum was compared to expected peaks based on differing activation modes. Energy peaks are isolated for half‐life measurements to find relative ratios and potential long‐term activation risk. Potential exposure to workers after multiple irradiation events was measured using a Ludlum 9DP and BeO OSLDs.

**Results:**

Expected radionuclide products are predicted depending on activation mode: Cu‐64, Cu‐66, Zn‐65, and Zn‐69 via neutron capture and Cu‐62, Cu‐64, Zn‐63, and Zn‐65 via photo‐neutron activation. Gamma‐spectrum analysis shows that photo‐neutron activation is the dominant source of exposure with decay via positron emission. The effective half‐life of the 511 keV positron annihilation peak is measured as 12.16 min, mostly from Cu‐62 and Zn‐63. Survey measurements indicate the dose to workers of the BMB to be 0.106 mrem per course of a 25 fx, 400 MU/fx, 15 MV treatment if the therapist handles the BMB every fx for 30 s. Using the OSLDs, under the same conditions but handling the bolus for 1 min per fraction, skin dose was estimated as 9.23 mrem per course.

**Conclusion:**

The dominant source of exposure from BMB is the result of photo‐neutron activation rather than neutron‐capture, as was historically assumed. Penetration of the resultant positron emission is considerable given the presence of annihilation photons compared to previously assumed beta decay and should be considered for exposure to workers.

## 1 INTRODUCTION

Brass‐mesh‐bolus (BMB) has been proposed as an alternative to traditional slab‐based water‐equivalent bolus due to its ease of setup and ability to conform to complex patient contours, attributed to its thin and flexible structure.^1^ A bolus is a material placed on the skin to increase surface dose during radiation therapy, compensating for tissue deficits. While water‐equivalent bolus materials have been the standard, brass mesh offers the advantage of being more adaptable to body contours, potentially improving treatment accuracy by reducing the risk of air gaps between the bolus and the patient's skin. Multiple studies have demonstrated that BMB has a similar dosimetric and clinical effect to water‐equivalent bolus in that it provides a similar dose distribution at the patient‘s surface, thereby justifying its adoption in many clinics.^1^, ^2^, ^3^, ^4^, ^5^, ^6^, ^7^

However, an overlooked risk emerges with BMB when used in high‐energy photon beams (typically 10 MV and above). Brass can undergo radioactivation when irradiated by these high‐energy beams. This poses a potential exposure risk to therapists due to cumulative radiation exposure. It could also provide a small amount of additional and untracked dose to the patient. While this phenomenon is acknowledged, little research has been done on the activation mechanism of BMB and the subsequent doses that result from realistic clinical usage. The assumption in previous literature has been that neutron capture—the absorption of neutrons by atomic nuclei, followed by radioactive decay—is the primary activation mechanism.^1^ This assumption necessitates further investigation. In similar fields of study, other research has demonstrated that metals, specifically hip prosthetics, are activated via the photo‐neutron effect far more than they are from neutron capture, giving reason to believe this might be the primary method for BMB as well.^8^

Only a singular reference, from Manger et al., was found that investigates the additional occupational dose to oncology staff when handling mesh bolus post‐treatment. This study calculates the anticipated activity produced via neutron capture actviation.^1^ Several assumptions are made in doing so, including the characteristics of the neutron fluence, which were taken from a paper reporting a fluence 40 cm out of field inferior to the isocenter. This discrepancy can be significant, as neutron fluence out of field is approximately 3.3 times less than at the isocenter.^9^, ^10^, ^11^ Activities are determined using a calculator designed for nuclear reactor‐based activation of metals.^12^ Photoneutron activation is presumed negligible; however, this is compared to the neutron dose to the patient rather than activation of the bolus itself, and therefore dose to staff or others is neglected. Additionally, this research is cited by the manufacturer for safety data information to warn users of the radiation safety risk of using higher energy beams (>10 MV).^13^ However, there are instances where these energies would be advantageous during treatment planning (e.g., tissue expanders), and the risk of using such beams should be weighed against well‐determined risk factors. Thus, this paper seeks to investigate occupational radiation safety for staff handling BMB using a more accurate understanding of the mechanisms of activation.

## 2 MATERIALS AND METHODS

To determine the expected activation of BMB, neutron‐capture products were calculated using thermal‐neutron cross‐section tables, a method established in previous literature.^1^ In addition, the study considered photo‐neutron activation components. Brass is an alloy typically made of copper and zinc, but its exact composition varies by product and is not provided by the manufacturer. It is assumed that common brass alloy compositions are used in manufacturing this product to predict the radionuclides likely to be produced through photoneutron interactions with the naturally occurring isotopes in these alloys.

**FIGURE 1:**
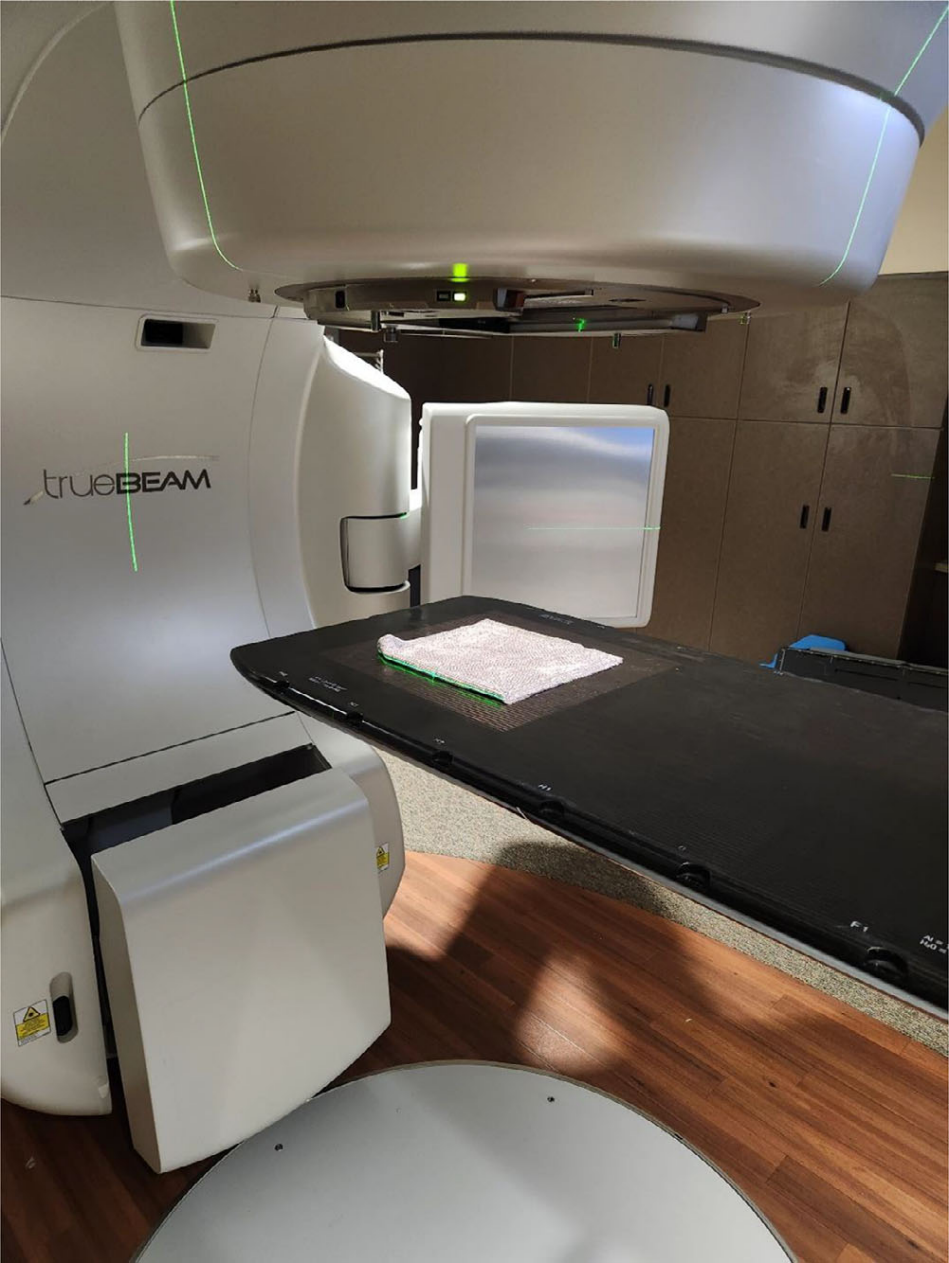
Experimental setup for in‐field BMB exposure. BMB folded in half twice to fit fully in the wide open field. BMB, brass‐mesh‐bolus.

To analyze the mechanisms of activation and properties of the resulting radionuclides, the painted BMB from Radiation Products Design, 5218 Barthel Industrial Drive, Albdertville, MN, item number 489–601, is irradiated by a Varian Truebeam linac under three primary conditions, 15 MV in‐field, 10 MV in‐field, and 15 MV out‐of‐field photon beams. In‐field beams are given a 30 × 30 cm square field with the BMB placed at isocenter, folded in half twice to fit fully within the field, as seen in Figure 1. For the out‐of‐field condition, conditions are unchanged except the BMB is placed 40 cm inferior from the isocenter and the jaws are closed to 2 × 2 cm to maximize neutron production and match setup conditions of previous neutron fluence research.^14^

Gamma spectrum measurements were taken using a ∼1.5 × 1.5 cm NaI(Tl) Picker Nuclear scintillator, a Gamma Spectacular GS‐PRO V5 spectrometer, and PRA spectrometry multi‐channel analyzer (MCA) software ^15^ with Interspec for spectrum analysis.^16^ The BMB was wrapped fully around the scintillator and placed between multiple lead aprons to lower the background noise during measurement. All gamma spectrum measurements were collected for a sufficient duration of time to resolve peaks with high statistical confidence. Gamma spectra are collected for all three irradiation conditions, 15MV in field and out of field and 10 MV in field.

By examining specific energy peaks in the gamma spectrum and comparing them to known gamma emissions from activated nuclides (e.g., Cu‐64, Zn‐65), radionuclides were identified as present or absent after irradiation.

To determine the specific half‐lives of the activated radionuclides in the BMB, distinct energy peaks were identified and isolated using the MCA software. The decay data, expressed as counts per minute (CPM), was tracked over the full duration of each measurement for each peak. For some energy peaks, contributions from multiple radionuclides were present, necessitating a more sophisticated analysis. In such cases, we used effective half‐life determination and multi‐component decay fitting, wherein the expected contributions from multiple radionuclides were modeled. By inputting the anticipated radionuclide ratios into a curve‐fitting algorithm, the software optimized the fit to the experimental decay data, allowing for the decomposition of overlapping decay signatures and the quantification of individual radionuclide contributions. This information was used to predict potential exposure to workers after multiple irradiation events, confirmed with measurements using a Ludlum 9DP placed at various distances from the bolus on a flat surface.

Dose measurements from the activated BMB are taken with two different methods. First, the BMB is placed flat on a countertop, and the dose is measured with a Ludlum 9DP pressurized ionization chamber placed directly on top of the BMB. Due to the thickness of the meter's chamber walls as well as the volume of the detector's active region, this is more equivalent to a deep dose measurement. To get a more accurate surface dose measurement, three low dose BeO personal dosimetry OSLDs (MyOSLchip system, Freidberg Instruments GmbH, Germany) are placed on the BMB directly, and the BMB is folded over the top of it as might be the case for a therapist having the BMB folded and draped over their hand while cleaning or removing it after treatment. Three more OSLDs are used for background subtraction. For both measurements, the BMB is irradiated with the 15 MV in field setup conditions, using a large number of MU to achieve good measurement statistics, and the measurements are scaled to match more realistic clinical conditions. Results are compared with occupational dose limits. Dose to a therapist is estimated by assuming a worst case scenario with usage of the BMB every day for a full 25 fraction course, as demonstrated by the intent of treatment for 76.5% of physicians internationally, as per an international survey performed by the Italian Association of Radiotherapy and Clinical Oncology.^17^

All measurements are taken after ensuring that the BMB has returned to near background activity relative to the measurement sensitivity. For dose/exposure measurements, this meant the pre‐irradiation exposure was equivalent to the background measurement. For gamma spectrometry measurements, this meant no significant peaks were observed pre‐irradiation during a measurement of similar measurement duration. Additionally, background measurements were frequently taken to ensure no significant differences were observed and to identify any possible background peaks that might not be related to the BMB.

## 3 RESULTS

**FIGURE 2:**
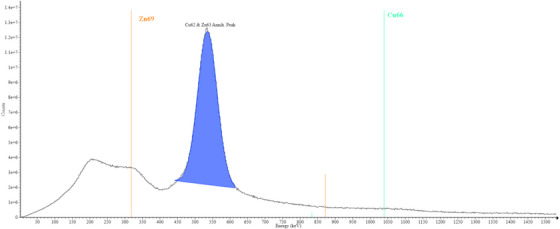
Gamma spectrum of BMB after irradiation in a 15 MV beam. Colored vertical lines indicate theoretical neutron‐capture peaks. The lack of peaks in these locations confirms activation is not due to neutron‐capture. BMB, brass‐mesh‐bolus.

**FIGURE 3:**
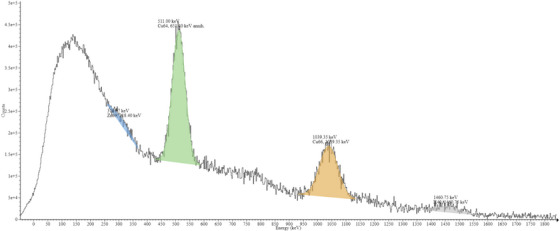
Gamma spectrum of BMB after irradiation out of field in a 15 MV beam. The Presence of expected peaks as predicted by neutron capture and the difference in spectral peaks appearing demonstrate that out‐of‐field interaction mechanisms are fundamentally different than in‐field interaction mechanisms, resulting in a different spectrum. BMB, brass‐mesh‐bolus.

Both photoneutron activation and neutron capture are probable during in‐field exposure of BMB. For the 15 MV gamma spectrometry results shown in Figure 2, a large 511 keV peak was observed, which is the predominant peak associated with photonuclear activation. While this peak is also expected for neutron capture, none of the additional peaks unique to Cu‐66 or Zn‐69 were observed. Notably, there is a lack of an observable Cu‐66 peak, which would be anticipated to contribute 96.7% of the total activity if neutron capture were the primary method of activation.^1^ Additionally, the out‐of‐field 15 MV gamma spectrum seen in Figure 3 demonstrates a different activation product emission spectrum compared to the in‐field irradiation of the BMB. The previously missing Cu‐66 peak is then clearly present, as anticipated for an out‐of‐field activation where neutron capture is the only probable activation mechanism. This discrepancy between the in‐field and out‐of‐field spectra suggests that the primary activation mechanism is due to the photoneutron effect rather than neutron capture.

**FIGURE 4:**
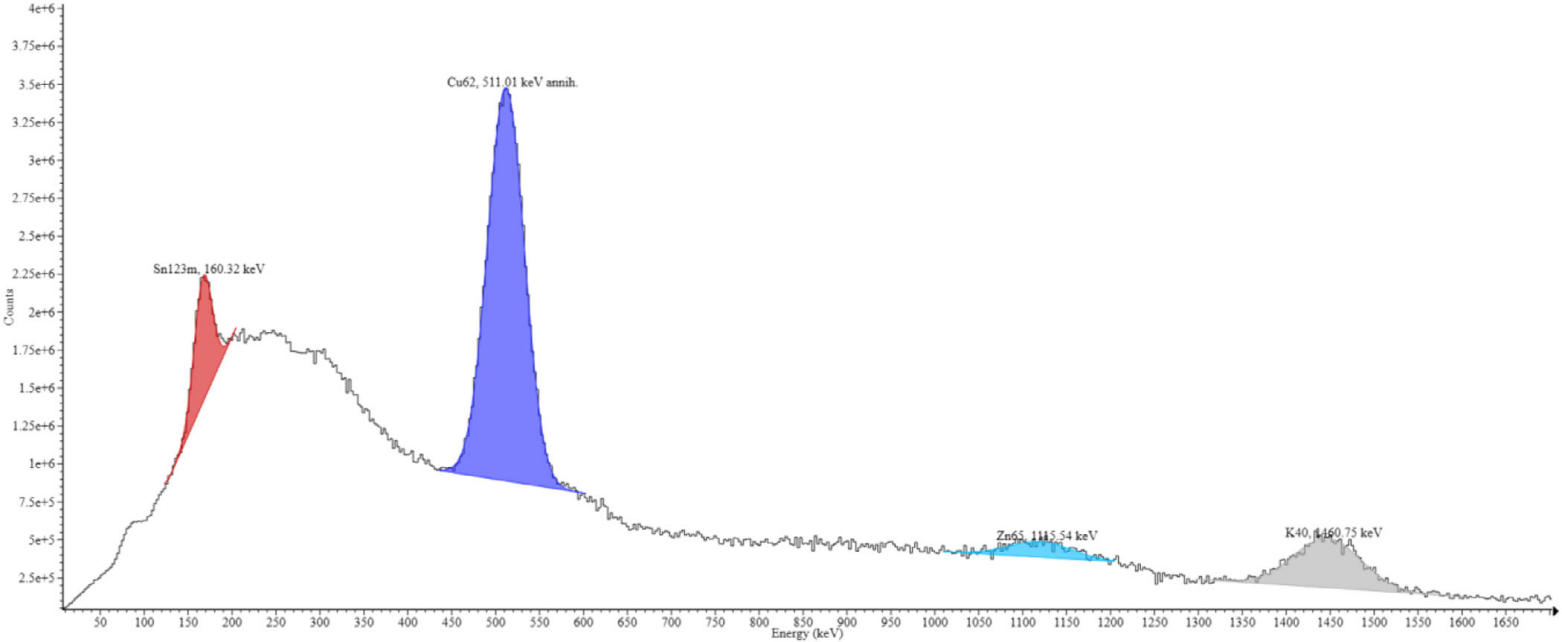
Gamma spectrum of BMB after irradiation in a 10 MV beam, demonstrating activation in medium energy fields. The presence of the 160 keV peak indicates Sn‐123 m in the brass alloy after irradiation due to photoneutron activation of Sn‐124. Different manufacturers and models may use different alloys, leading to varying risks to therapists. BMB, brass‐mesh‐bolus.

The 10 MV gamma spectrometry results shown in Figure 4 showed a 160 keV peak associated with Sn‐123 m, indicating that the composition of BMB is not a typical alloy and the composition might impact the activation products and therefore the dose. Specifically, it appears that the activation rate of tin is very low, so a yellow brass (e.g., unpainted BMB) that contains much less, if not any, tin per unit mass would therefore have relatively more copper/zinc per unit mass than that in white brass (e.g., painted brass). This would result in higher activation in 15 MV beams. Unfortunately, the exact composition is neither standardized nor disclosed by the manufacturer, which may limit the understanding of potential dose risks.

Our measurements showed that in‐field BMB activation was 27 times higher than out‐of‐field activation, suggesting that the majority of activation occurs when BMB is directly exposed to the primary radiation beam. Based on previous neutron fluence study results, 9 10 the neutron fluence ratio between isocenter and 50 cm inferior is approximately 3.3, suggesting that neutron capture is not the dominant effect but rather the photonuclear effect is the major contributing factor for the discrepancy of activation in field versus out of field.

**FIGURE 5:**
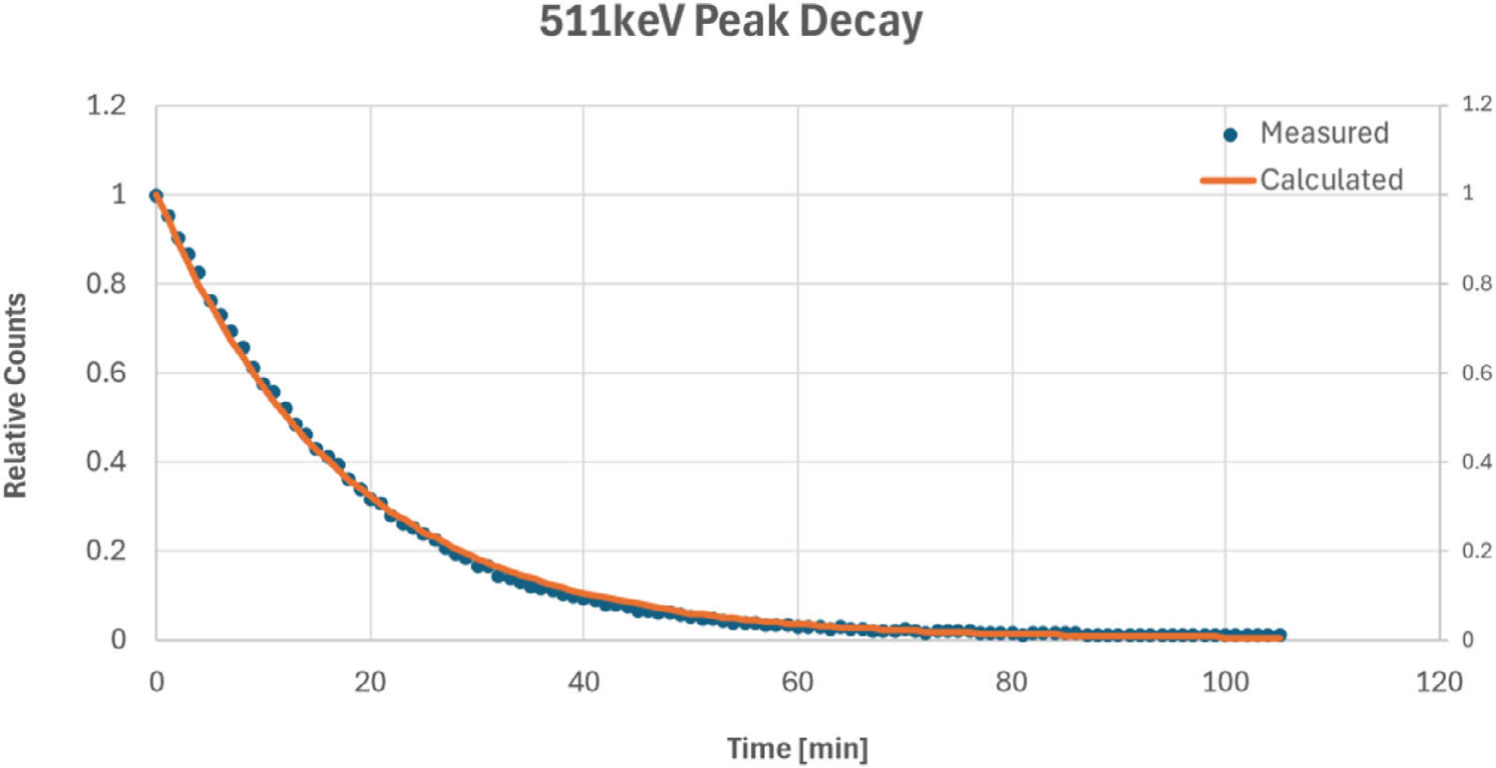
Scintillator measured half‐life of 15 MV beam‐activated BMB, windowed on the 511 keV energy peak, with an effective half‐life of 12.16 min. Cu‐62 and Zn‐63 are the anticipated radionuclides, with Cu‐62 contributing approximately 87% and Zn‐63 13% to the activity. BMB, brass‐mesh‐bolus.

**FIGURE 6:**
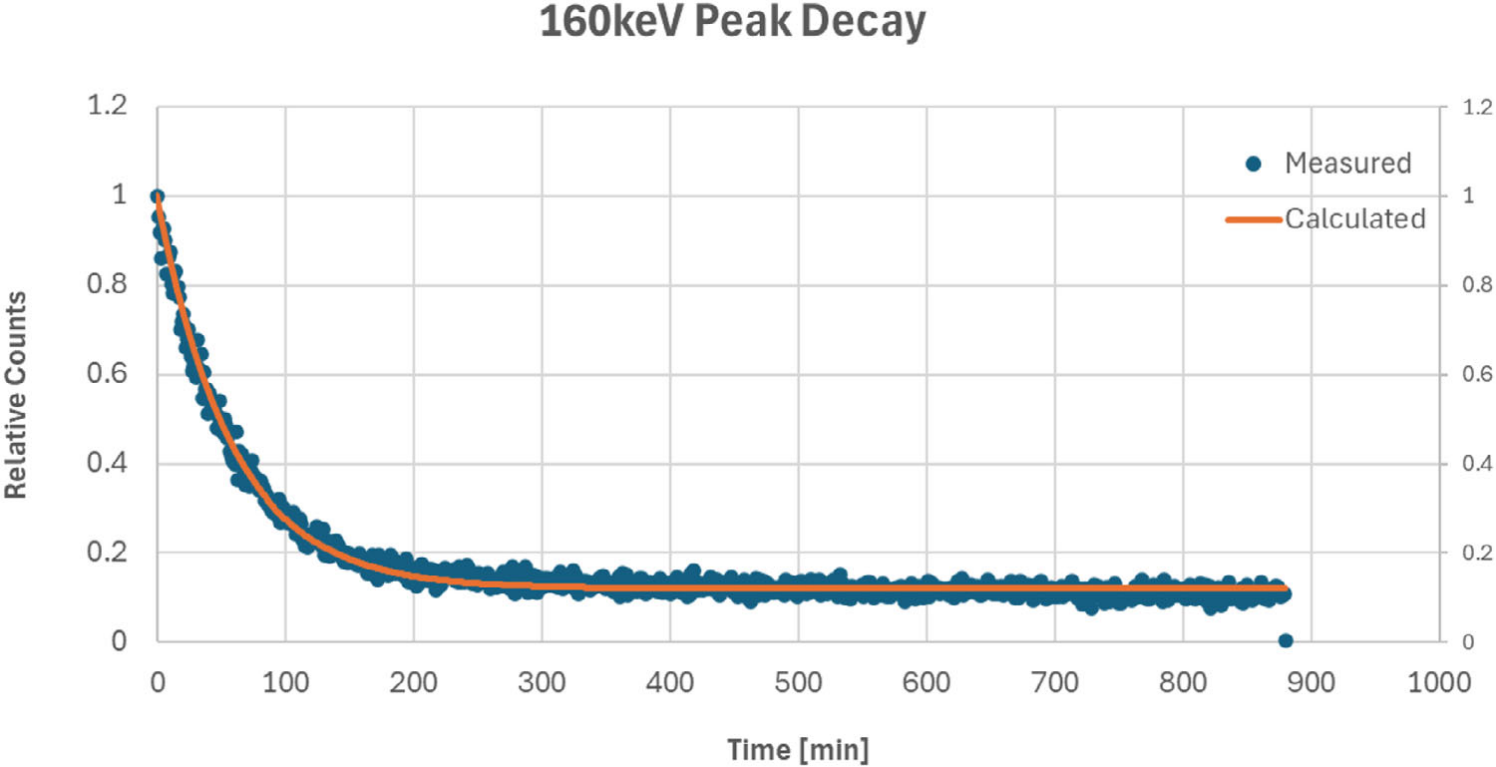
Scintillator measured half‐life of 10 MV beam‐activated BMB, windowed on the 160 keV energy peak, closely matching the decay of Sn‐123 m with a measured half‐life of 40.17 min. BMB, brass‐mesh‐bolus

The 511 keV peak from the 15 MV beam activation had a measured half‐life of 12.16 min, as shown in Figure 5, which aligns more closely with decays from Cu‐62 and Zn‐63 rather than Cu‐64′s half‐life of 12.7 h. Performing a multi‐component decay fitting with a mixed source decay model considering Cu‐62, Cu‐64, and Zn‐63 yields an approximate ratio of 87% Cu‐62, 0% Cu‐64, and 13% Zn‐63 with a curve fitting R^2^ of 0.058. The 160 keV peak from the 10 MV exposure shown in Figure 6 had a measured half‐life of 40.17 min, which agrees within 0.2% of the half‐life of Sn‐123 m. This peak did not show up in the 15 MV irradiation due to the vastly higher activation rate of the copper once the energy thresholds of activation, which based on the data appear to be somewhere between 10 and 15 MV, are achieved and the signals from the Sn‐123 m are drowned out by the orders of magnitude greater signal from the copper radionuclides.

Table 1 provides dose measurements for various distances from the bolus using a Ludlum 9DP. For the closest measurement to the BMB, the dose to the therapist while handling was measured at 1.70 mrem/h‐per‐1000 MU. This was determined by irradiating the BMB to 1000 MU and immediately placing the survey meter directly on the bolus. By scaling this measurement linearly to 500 MU, assuming the exposure is repeated 25 times for a full course of treatment, and also assuming the therapist handles the bolus for 1 min per fraction, the estimated potential dose to a therapist is 0.354 mrem per 25 fraction course, as a deep dose measurement. However, using an OSLD to achieve a surface dose measurement demonstrated a much higher potential dose under similar conditions. Using the same exposure conditions and then folding the OSLD in the bolus with 2 h of contact was measured to be 17.13 mrem. Using the previously measured decay curve to estimate dose during the first 1 min, which calculates to 5.39% of the 2 h total dose being delivered in the first 1 min, and using the same handling assumptions and scaling as before, the skin dose was measured to be 11.54 mrem. This is assuming each fraction the BMB has time to fully decay overnight, which, if there are multiple fractions of 15 MV treatments back‐to‐back, the dose could be potentially higher due to activation buildup.

**TABLE 1:** Dose to therapist/fetus after handling BMB.

Distance (cm)	Exposure rate per MU (mR/h per MU)	Dose to therapist per course (mrem)
5	0.0017	0.3539
10	0.0011	0.2254
20	0.0005	0.1107
30	0.0004	0.0769
40	0.0003	0.0522

*Note*: Dose measurements were corrected to the time immediately after 1000 MU 15 MV exposure for differing distances from the bolus measured with a Ludlum 9DP pressurized ionization chamber. Distance: Measurement from BMB surface to center of 9DP measurement volume. Exposure Rate per MU: Estimated exposure rate acquired per MU delivered from activation. Dose to Therapist per Course: Assuming a standard course of 25 fx (500 MU/fx) with the same therapist holding the BMB for 1 min every fx.

Abbreviation: BMB, brass‐mesh‐bolus.

## 4 DISCUSSION

The gathered data suggests that the previously assumed activation methods for BMB, and thus potential risks in handling, have been mischaracterized. Unlike brass neutron capture activation products, photonuclear activation products generate large photon yields mixed with dose from positrons that behave like electrons in range and dose deposition. Therefore, this additional dose may be missed by standard personal dosimeters and thick walled detectors such as pressurized ionization chambers, which could lead to greater radiation safety concerns for skin dose. Additionally, the painted BMB used in this study appears to be comprised of a white brass alloy, containing large quantities of tin as demonstrated by the Sn‐123 m decay peak. This radionuclide does not pose as great a risk as copper or zinc due to its relatively small photonuclear cross‐section and lower decay energy. In a yellow brass, that is, unpainted BMB from the same supplier, the surface dose may be higher than found in this study due to a higher concentration of copper and zinc. We did not have the unpainted BMB for this study, and so this remains to be tested experimentally.

In comparing our results to Manger et al., we measured somewhat comparable dose rates using an ionization chamber, with Manger's result demonstrating 0.4 mrem/h after a 500 MU irradiation and ours showing 1.7 mrem/h after a 1000 MU irradiation. Scaled to match the 500 MU irradiation conditions, this is 0.85 mrem/h. However, using an OSLD demonstrated a potential for a higher skin dose of 11.54 mrem per course, or 27.7 mrem/h immediately after irradiation of 500 MU.

Comparing the doses observed to occupational dose limits, this would be only 0.007% of the total effective dose equivalent limit using the ionization chamber readings and 0.023% of the extremity dose limit using the OSLD readings for one course. Given the number of assumptions made, this needs to be reconsidered by the site physicist for their own clinic's workloads and bolus usage, as it does not account for activity buildup, multiple courses, different energies, bolus composition, different bolus handling habits, and so forth.

It is crucial to verify any assumptions regarding radiation protection to ensure the safety of staff. Therapists should minimize contact with the BMB post high‐energy (15 MV and above) photon treatments, adhering to ALARA (As Low As Reasonably Achievable) principles. While the anticipated dose is likely small, it is the responsibility of the physicist to understand all potential sources of exposure to patients and staff and inform therapists as to how to mitigate easily avoidable exposure through conscientious BMB handling habits following treatment. It should also be noted that common badging practices may not identify this increased level of exposure unless therapists are equipped with ring badges as they are handling the BMB.

## 5 CONCLUSIONS

The dominant source of exposure from BMB arises from photo‐neutron activation rather than neutron capture. This finding has important implications for radiation safety protocols, especially for therapists who frequently handle BMB, such as during setup and patient positioning. Photo‐neutron activation of BMB results in large fractions of positron decays, giving it a high photon emission fraction and average photon energy, along with the risk of increased hand dose that would not be measured by typical occupational radiation badges. While the presence of annihilation photons is not a new discovery given the previously assumed presence of Cu‐64, neutron‐activation would have resulted in 97% of the activity occurring because of Cu‐66 and only 0.0008% from Cu‐64, whereas photo‐neutron activation appears to result in the majority of activation occurring from Cu‐62 and Zn‐63, both positron emitters. Adherence to the ALARA principle remains critical to minimizing unnecessary exposure, which can be easily avoided with basic knowledge of the activation risk and methods to minimize contact with the BMB or avoid activation entirely by limiting usage of 15 MV or above beams on the BMB.

## AUTHOR CONTRIBUTIONS

Richard C. Mallory: Functioned as the primary investigator, performing measurements and primarily authoring the paper. Kyle Woods: Contributed by supervising and advising direction of the research and writeup. Kiernan McCullough: Contributed by supervising and advising direction of the research and writeup.

## CONFLICT OF INTEREST STATEMENT

The author declare no conflicts of interest.
